# Valorization of saffron (*Crocus sativus* L.) stigma as a potential natural antioxidant for soybean (*Glycine max* L.) oil stabilization

**DOI:** 10.1016/j.heliyon.2024.e25875

**Published:** 2024-02-07

**Authors:** Moussa Nid Ahmed, Karima Abourat, Jamila Gagour, El Hassan Sakar, Khalid Majourhat, Jamal Koubachi, Said Gharby

**Affiliations:** a Biotechnology, Analytical Sciences and Quality Control team Faculty Polydisciplinary of Taroudant, University Ibn Zohr, Morocco; b Laboratory of Biology, Ecology and Health, FS, Abdelmalek Essaadi University, Tetouan, Morocco; c Geo-Bio-Environmental Engineering and Innovation Laboratory, Molecular Engineering, Biotechnology and Innovation Team, Polydisciplinary Faculty of Taroudant, University Ibn Zohr, Agadir, Morocco

**Keywords:** Saffron stigmas, Natural antioxidant, Soybean oil, Oxidative stability, Multivariate analysis

## Abstract

Synthetic antioxidants are known for their efficiency to improve vegetable oil oxidative stability. But owing to their harmful effects on human health, edible oil industry is seeking for safe and healthy natural antioxidants. The present work was setup with the aim of improving soybean oil (SO) oxidative stability by using saffron (*Crocus sativus* L.) stigmas collected in Morocco. Saffron stigmas were used as a natural antioxidant at various concentrations (0.2, 0.3, and 0.6%) in soybean oil compared to tocobiol (0.3%) as a synthetic antioxidant (the positive control). Performances of such natural and synthetic antioxidants were evaluated by measuring oil basic quality indices under accelerated storage at 60 °C for 12 weeks. Such indices consisted of free fatty acids (FFA), peroxide value (PV), anisidine value (p-AV), total oxidation value (TOTOX), UV extinction coefficients (K232 and K270), fatty acids composition (FA), and iodine value (IV). The obtained data show that there were significant (p < 0.05) increases in FFA, PV, p-AV, K232, K270, and TOTOX but no much variations were observed for FA and IV especially in saffron stigmas fortified oils across storage times. However, in the case of oils fortified with saffron stigmas at different doses, such an increase was of a lesser magnitude (for FFA, PV, p-AV, K270, and TOTOX) as compared to tocobiol. These outcomes were confirmed by principal component analysis with strong positive correlations (p < 0.001) among FFA, PV, p-AV, K232, K270, and TOTOX. The most important, for which determination coefficient R^2^ > 0.9, were modeled through simple regressions. In conclusion, saffron stigmas with the different doses performed better than the positive control (tocobiol) regardless of the storage time. It could be concluded that saffron stigmas are a promising natural antioxidant, alternative to synthetic antioxidants, to enhance the oxidative stability of edible oils.

## Highlights

• *Crocus sativus* L. stigmas (CSS) were valorized as a natural antioxidant.
• CSS induced ameliorative effects of Glycine max L. oil (GMO) properties.
• CSS ameliorative effects on GMO were dose dependent as a general trend.
• GMO basic quality indices and fatty acids were monitored for 12 weeks.
• CSS had the potential as alternative for synthetic antioxidants.

## Graphical abstract

**Figure:**
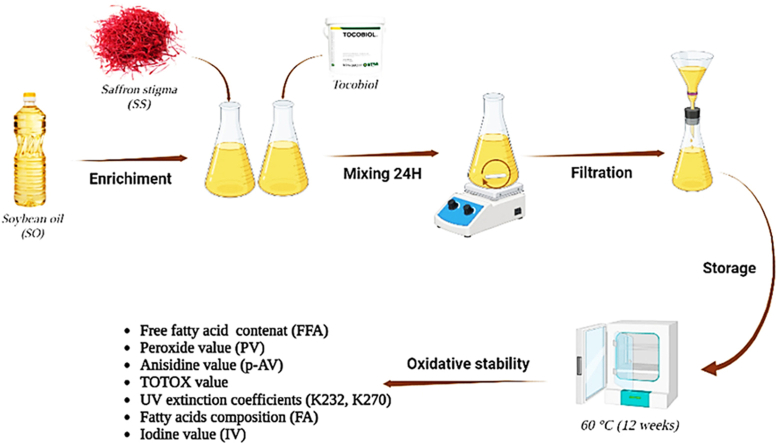

## 1 Introduction

The bottleneck of vegetable oils’ conservation is their sensitivity to oxidation [1]. Yet, lipid oxidation process is a complex phenomenon involving several reactions that begin immediately after oil extraction [2]. It occurs further during processing, storage, shipping, and final formulation of lipid-based foods [3]. From a nutritional stand point, lipids contain numerous compounds of crucial importance for human nutrition [4], however, their oxidation is the main cause of their quality deterioration [5]. Its progression leads to the degradation of proteins and lipids resulting in the deterioration oil taste, color, and texture [6]. Such changes are associated to the occurrence of rancid odors and flavors that alter and reduce the nutritional quality of food [7], even going so far as to generate chemical compounds that are harmful to human health [8].

Oils and fats are subject to oxidation processes involving three main interaction pathways namely auto-oxidation, photo-oxidation, and enzymatic oxidation [4,9]. Speed of such an oxidation depends predominantly on the lipid composition and the storage conditions. Autoxidation is induced by autocatalytic reactions of free radicals or triplet oxygen (^3^O_2_) [10]. It is based on a chain of radical reactions involving three phases: Initiation, propagation, and termination [11]. However, photo-oxidation is promoted by UV light or singlet oxygen (^1^O_2_) [10]. It remains the most dangerous since it leads to the formation of hydroperoxides without formation of radicals, and proves to be, at least 1000 to 1500 times, faster than auto-oxidation and enzymatic oxidation [12]. This latter, as its name suggests, relies on the presence of moisture and oxidizing enzymes such as lipoxygenases, cyclooxygenases, esterases, and cytochromes P450 [10,13].

Soybean (Glycine max L.) or soy is ranked among the world's most consumed polyunsaturated vegetable oils [14], and the second most produced oil worldwide after palm oil [15]. Originated from East Asia, soybean is an oilseed crop widely cultivated mainly for table oil production. Its production and exports are dominated by the United States, where the harvested area increased from 0.27 Mha in 2008 to 0.98 Mha in 2018 [16]. Based on some estimations, Argentina and Paraguay will produce 66 and 12 million tons of soybeans, respectively by 2027 [16]. Such production increase of soybean is justified by its nutritional value as well as the global market demand. Soybeans are rich in omega-3 (linolenic acid) polyunsaturated fatty acids [17], and are an excellent source of quality proteins, dietary fiber, and isoflavones [18]. In addition, soybeans contain a multitude of vitamins as well as 15 essential minerals such as potassium, iron, phosphorus, and calcium [19,20]. In terms of fatty acid profile, soybean oil (SO) contains 25% oleic acid, 55% linoleic acid, and 8% linolenic acid [21]. Despite this good nutritional profile (high content of unsaturated fatty acids), SO is very sensitive to oxidation. Indeed, this particularity of the presence of large quantities of linoleic and linolenic acids, which are sensitive to oxidation, reduces its oxidative stability [15,17]. As a result, the organoleptic properties of SO and its derived products are altered.

To solve such an issue related to oxidation and instability of edible vegetable oils (EVOs), food industry resorts to incorporate antioxidants into EVOs [14]. The added antioxidants have a positive effect on EVOs’ quality, fats and foods containing fats by maintaining their nutritional values [22]. Two types of antioxidants are basically applicable namely natural and synthetic. The most important synthetic antioxidants are BHA (butylated hydroxyanisole), BHT (butylated hydroxytoluene), PG (propyl gallate, propyl 3,4,5-trihydroxybenzoate), and TBHQ (tert-butylhydroquinone) [23,24]. They are primary antioxidants [5]. However, despite their ability to increase the oxidative stability of EVOs, synthetic antioxidants have been reported, in many studies, to have carcinogenic side effects [25]. Thus, there is great interest today in replacing synthetic antioxidants with natural antioxidants [23]. Natural compounds are healthier than synthetic ones [26]. In this regard, various recent studies have highlighted the benefits of using natural antioxidants, originating from plants, to improve the stability of vegetable oils [6,9,17,25,27,28].

Saffron, dried red stigmas obtained from the flowers of the cultivated plant *Crocus sativus* L., is one of the world's best-known medicinal and aromatic plants [29,30]. It is known as "red gold" [30,31]. In its composition, saffron stigmas (SS) have more than 150 volatile, non-volatile, and aromatic compounds [32]. These include carotenoids, flavonoids, vitamins (riboflavin and thiamine), anthocyanins, amino acids, and proteins [33]. The stigmas are also distinguished by four main biologically active constituents: Safranal, picrocrocin, crocetin, and crocin [34,35]. As such, they may have important pharmacological properties including antioxidant activity. It is a rich source of antioxidants [36]. A large number of these compounds, such as crocetin, crocins, phenolic compounds, and flavonoids, give saffron its antioxidant properties [36]. Zhang et al. [37], revealed that saffron offers a readily available source of natural antioxidants, which can serve as food additives as well as nutritional supplements. High content of antioxidant compounds makes of SS a good nutraceutical [38].

A great deal of research has been conducted over the past two decades on the application of plant extracts endowed with an important antioxidant capacity for the fortification and stabilization of EVOs [7]. Our literature synthesis revealed a lack of documented research concerning the application of *C. sativus* stigmas as a natural source of antioxidants to improve SO oxidative stability. To fill this knowledge gap, the present study was undertaken to investigate the potential antioxidant impact of *C. sativus* stigmas on SO oxidative stability in comparison with both untreated SO and SO fortified with a synthetic antioxidant (tocobiol).

## 2 Material and methods

### 2.1 Plant material sampling

Refined soybean oil free of synthetic antioxidants was provided by the ‘Huileries du Souss Belhassan, HSB” company (Agadir province, Morocco). In the 2021/2022 crop season, at the full flowering stage, saffron stigmas were collected from local saffron groves in Taliouine town (30°31′48″N, 7°55′29″W, 1586 m.a.s.l) known as the saffron production center in Morocco. Saffron stigmas were dried in a ventilated oven at 105 °C for at least 3h until reaching a constant weight. The obtained dry biomass was then grounded into a fine powder, which was kept at 4 °C prior to soybean oil enrichment.

### 2.2 Reagents and chemicals

All chemicals and reagents used in this study are of analytical grade (Sigma Aldrich). The main reagents and chemicals used are sodium hydroxide, potassium hydroxide, sodium chloride, potassium iodide, anisidine reagent, anhydrous sodium sulfate, ethanol, isooctane, acetic acid, methanol, cyclohexane, chloroform, and sodium thiosulfate. Tocobiol was as used in this study as a synthetic antioxidant; it was provided by the above indicated company HSB.

### 2.3 Experimental setup

The experimental design used consisted in a repeated random complete design with three replicates for each measurement. Different doses of saffron stigma powder (0.2, 0.3, and 0.6% w/w) were introduced separately into dark glass flasks containing soybean oil (SO). Similarly, 0.3% of tocobiol was prepared as a positive control, while the negative control (NC) consisted in SO only without any additives. The preparations were then kept under agitation in darkness for 24h. The obtained enriched oils were then filtered using a filter paper. The enriched oils obtained after filtration were labeled as follows: SO enriched with SS at 0.2% (S–S0.2), 0.3% (S–S0.3), and 0.6% (S–S0.6) against the positive control namely SO fortified with tocobiol at 0.3% (S-T0.3). Finally, all obtained samples (fortified SOs along with the NC) were subjected to an accelerated storage at 60 °C to evaluate the evolution of its oxidation state for a period of 12 weeks.

Physicochemical quality of all oil samples was monitored by determining a set of parameters. These include free fatty acids (FFA), peroxide value (PV), anisidine value (p-AV), TOTOX value, and UV extinction coefficients (K232 and K270). These parameters were measured two weeks throughout the storage period (12 weeks of storage at 60 °C). Fatty acid composition (FA) together with iodine value (IV) were determined at the beginning and at the end of the experiment.

### 2.4 Soybean oil physicochemical traits

#### 2.4.1 Free fatty acids (FFA)

FFA content of different SO samples was measured according to the official ISO method (ISO 660, 2020) [39]. A test weight consisting of 10 g of oil was dissolved in ethyl alcohol and titrated with NaOH solution (0.1 N) in the presence of phenolphthalein as an indicator. The result was expressed as percentage of oleic acid (% w/w).

#### 2.4.2 Peroxide value (PV)

PV was determined using the method specified in (ISO 3960, 2017) [40]. In this method, 5 g of oil sample were dissolved in an acetic acid/chloroform mixture (3:2) and subsequently reacted with a supersaturated potassium iodide (KI) solution. After allowing the reaction to proceed for 5 min in darkness, the mixture was titrated with a 0.01 N sodium thiosulfate solution (Na_2_O_3_S_2_) in the presence of starch as an indicator. The outcome was expressed as milligrams of peroxide per kg of oil (mEq O_2_/kg oil).

#### 2.4.3 Anisidine value (p-AV)

The p-AV values were measured using the normalized method described in standard (ISO 6885, 2016) [41]. 2 g of oil were dissolved into 25 mL of isooctane and the absorbance was measured at 350 nm with isooctane as a blank. 5 mL of the solution were taken and mixed with 1 mL of p-AV reagent (0.25%, w/v glacial acetic acid). After 10 min of reaction, the absorbance was measured read at 350 nm with the isooctane/anisidine reagent mixture (5:1, v/v) as a blank. Absorbance was measured using a dual beam UV–Vis spectrophotometer (Shimadzu UV-1800). The p-AV was determined as follows **(**Eq. (1)**)**:

(1) $$p-\mathrm{AV}=\frac{25\;\left(1.2{\;A}_{s}-A_{b}\right)}{m}$$

Where, p-AV: *p*-anisidine value, A_s_: Absorbance of oil solution after reaction with *p*-anisidine, A_b_: Absorbance of the unreacted test solution, m: Mass of test sample in g, 25 is the volume of isooctane used to dissolve the oil, and 1.2 is a correction factor for dilution of sample solution with 1 mL of *p*-anisidine solution.

#### 2.4.4 Total oxidation value (TOTOX)

TOTOX is determined by combining the PV (which determines the primary oxidation products) and the p-AV (which measures the secondary oxidation compounds) according to the following formula **(**Eq. (2)**)** [42]:

(2) TOTOX = 2 × PV + p-AV

#### 2.4.5 UV extinction coefficients (K232 and K270)

K232 and K270 were determined as UV extinction coefficients according to the method described in (ISO 3656, 2011) [43]. The K232 and K270 values represent specific absorbance coefficients at their respective wavelengths. These coefficients are obtained by measuring the absorbance of an oil sample at specified wavelengths namely λ = 232 nm (conjugated dienes, CDs) and λ = 270 nm (conjugated trienes, CTs). To this end, 0.25 g of oil is dissolved into 25 mL of cyclohexane and the absorbance of mixture is read at 232 and 270 nm by dual beam UV–Vis spectrophotometer (Shimadzu UV-1800) with 1 cm as a path length. Cyclohexane was used as a blank sample.

#### 2.4.6 Fatty acid (FA) composition

FA composition of the samples was evaluated by gas chromatography according to the standard (ISO 12966-2, 2017) [44]. FA methyl esters were prepared by mixing 0.1 g of oil, 2 mL of isooctane, and 0.1 mL of methanolic potassium hydroxide (2 N). The mixture was well shacked for 1 min and allowed to stand for 2 min which was then washed with 2 mL of saline sodium chloride solution (40%). Then, the organic top layer was dried over anhydrous sodium sulfate. Esters of fatty acids were identified by HP-chromatograph (Agilent, USA) equipped with DB 23 AG-TRANS capillary column (60.0 m × 320 μm × 0.25 μm nominal) and a flame ionization detector. Helium was used as a carrier gas with a flow rate of 20 mL/min. The temperature of the oven, the injector, and the detector were set at 260 °C. Results were expressed based on the relative percentage of areas.

#### 2.4.7 Iodine value (IV)

IV is used to evaluate the vegetable oil unsaturation degree. It is defined as the weight of iodine absorbed per 100 g of oil or fat [45]. IV was calculated from the percentages of unsaturated fatty acids according to the formula **(**Eq. (3)**)** [46]:

(3) IV = (%C16:1 × C_O1_) + (%C18:1 × C_O2_) + (%C18:2 × C_O3_) + (%C18: 3 × C_O4_)

Where: C16:1: Palmitoleic acid, C18:1: Oleic acid, C18:2: Linoleic acid, C18:3: Linolenic acid. C_O1_ (1.001), C_O2_ (0.899), C_O3_ (1.814), and C_O4_ (2.737) are the iodine value coefficients of each corresponding fatty acid.

### 2.5 Data statistical analysis

The results were expressed as mean values ± standard deviation (SD, n = 3). The least significant difference (LSD) test was used for mean values separation and the differences were deemed significant at p < 0.05. Multivariate analysis (MA) was performed on data mean values for routinely measured oil quality indices monitored throughout the storage period (FFA, PV, p-AV, K232, K270, and TOTOX value). Before running MA, the data were checked for normality using Kolmogorov–Smirnov test. Such MA includes Pearson correlations study, simple regression modeling, and principal component analysis. Mean values’ graphs were drawn using OriginPro software (version 9.1, OriginLab Inc., USA), while MA associated graphs were created through Statgraphics package version XVIII (Statpoint Technologies, Inc., Virginia, USA).

## 3 Results and discussion

### 3.1 Changes in FFA

**Fig. 1:**
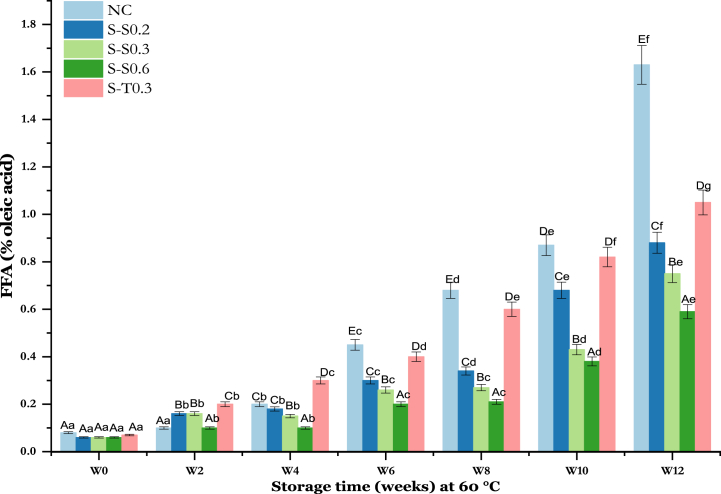
Changes in FFA of soybean oil (SO) fortified with saffron stigma (SS) during 12 weeks of storage at 60 °C. **NC**: Negative control, **S–S0.2**: SO + 0.2% SS, **S–S0.3**: SO + 0.3% SS, **S–S0.6**: SO + 0.6% SS, **S-T0.3**: SO + 0.3% tocobiol. Different letters represent significant differences (p < 0.05) between the oils. The uppercase letters correspond to different treatments for each storage period, while the lowercase letters correspond to the storage periods (W0 to W12) for each treatment.

FFA have a major influence on the oxidation of oils [47]. Measurement of FFA allows an examination of the oil degradation rate [48]. In other terms, FFA reflect the quantity of fatty acids hydrolyzed from triacylglycerols [49]. Enriched and negative control oils were analyzed for FFA content at 60 °C for 12 weeks of storage. Fig. 1 shows the variation in FFA content (% oleic acid) for the negative control (NC), saffron-enriched oils (S–S0.2, S–S0.3, S–S0.6) and tocobiol-enriched oil (S-T0.3) as a positive control.

The initial FFA content exhibited comparable values (p > 0.05) across all studied oils. Thereafter, a significant increase was observed, during the accelerated storage period (12 weeks, 60 °C). In particular, soybean oil enriched with 0.3% tocobiol showed the highest increase (p < 0.05) especially after the 6th week of storage. The negative sample (NC) showed similar levels of significance to soybean oil enriched with 0.2% saffron stigma (SS). Soybean oil enriched with 0.6% saffron stigma (S–S0.6) showed the slowest progression over this period. Consequently, a substantial and continuous rise was observed in the negative control (NC) throughout the storage period, reaching a higher value (1.63 ± 0.01%) and an increase 20 times greater than the initial state (0.08 ± 0.01%). This FFA increase over the storage period was significantly higher than that of saffron-enriched oils for lower SS doses (S–S0.2, S–S0.3, and S–S0.6). These oils demonstrated a slower FFA dynamic depending upon the antioxidant doses, ranging from 0.6 ± 0.01 to 0.88 ± 0.02% (S–S0.2), 0.06 ± 0.01 to 0.75 ± 0.03% (S–S0.3), and 0.06 ± 0.01 to 0.59 ± 0.02% (S–S0.6). The application of the tocobiol antioxidant reduced the evolution of oil FFAs but at a lesser extent than that observed with the three doses of SS. This is reflected in an increase in S-T0.3 FFA (0.07 ± 0.01–1.05 ± 0.02%). These results indicate an excellent performance of SS (a natural antioxidant) at various doses (0.2, 0.3, and 0.6%) in protected SO from hydrolysis better than tocobiol (a synthetic antioxidant). As the storage time increased, the FFA content increased. This is due to the hydrolysis of the ester bonds of triacylglyceride molecules under the effect of heat [50]. Javidipour et al. [50], noted a significant increase in FFA levels (p < 0.05) in hazelnut, olive, soybean, and sunflower oils during exposure to microwave heating. Analysis of FFA levels of table oils after the incorporation of different plant extracts have been the subject of various studies [[51], [52], [53]]. The results of these studies showed that natural antioxidants from plant extracts were better at protecting the oils from hydrolysis than synthetic antioxidants and the negative control, which is in accordance with our results.

### 3.2 Primary oxidation

#### 3.2.1 Changes in PV

**Fig. 2:**
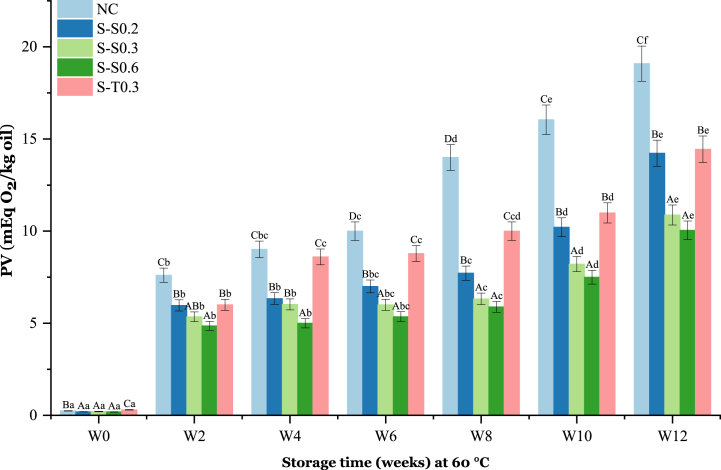
Changes in PV of soybean oil (SO) fortified with saffron stigma (SS) during 12 weeks of storage at 60 °C. **NC**: Negative control, **S–S0.2**: SO + 0.2% SS, **S–S0.3**: SO + 0.3% SS, **S–S0.6**: SO + 0.6% SS, **S-T0.3**: SO + 0.3% tocobiol. Different letters represent significant differences (p < 0.05) between the oils. The uppercase letters correspond to different treatments for each storage period, while the lowercase letters correspond to the storage periods (W0 to W12) for each treatment.

PV is an indicator [54] of the initial stages of oxidative change [55,56]. A chemical factor used to measure the degree of rancidity of oils. PV values, above 9 mEq O_2_/kg oil, indicates an oil oxidation [57]. Changes in the PV values of the oil samples are presented in Fig. 2.

The initial PV of all samples was similar (about 0.20 mEq O_2_/kg oil). After the first week of storage, PV increased sharply to 7.6 ± 0.39, 5.97 ± 0.31, 5.35 ± 0.30, 4.86 ± 0.31, and 6 mEq O_2_/kg for NC, S–S0.2, S–S0.3, S–S0.6, and S-T0.3, respectively (Fig. 2). A slight increase in PV was then observed for all samples and for all storage time intervals. At the end of 12 weeks of storage, PV levels reached 14.23 ± 0.51, 10.88 ± 0.55, 10.05 ± 0.42, and 14.4 ± 0.58 mEq O_2_/kg oil for S–S0.2, S–S0.3, S–S0.6, and S-T0.3 SO, respectively. The PV increased in SO samples from the beginning to the end of the storage period showing a linear progression of oxidation. The PV increase can be attributed to the formation of hydroperoxides, primary oxidation products [58]. The oil samples treated with the natural antioxidant (SS) especially S–S0.6 had significantly (p < 0.05) lower PV values than the negative control oil. This shows that the natural antioxidants in SS prevented the formation of primary hydroperoxides better than the synthetic antioxidants and thus protected SO from oxidation more effectively. SS contains crocetin which is an effective lipid peroxidation inhibitor and a free radical scavenger [31]. It also contains safranal and crocin which are able to eliminate free radicals [31]. Shanker et al. [9], showed that purslane (*Portulaca oleracea* L.) leaf extract (500, 1000, and 1500 ppm) and the antioxidant TBHQ (100 ppm) have a greater ability to prevent an increase in PV of SO when heated at 30 ± 2 °C for 5 days. Likewise, Okhli et al. [59], showed that the PV, measured after adding citron peel (*Citrus medica* L.) essential oil to sunflower oil, was similar to the addition of BHT after 3 days of storage at 65 °C. In recent years, Hassan et al. [55], showed that thyme, sage, rosemary, and TBHQ reduced significantly (p < 0.01) the increase in PV compared with the negative control that had the highest value.

#### 3.2.2 Changes in K232

**Fig. 3:**
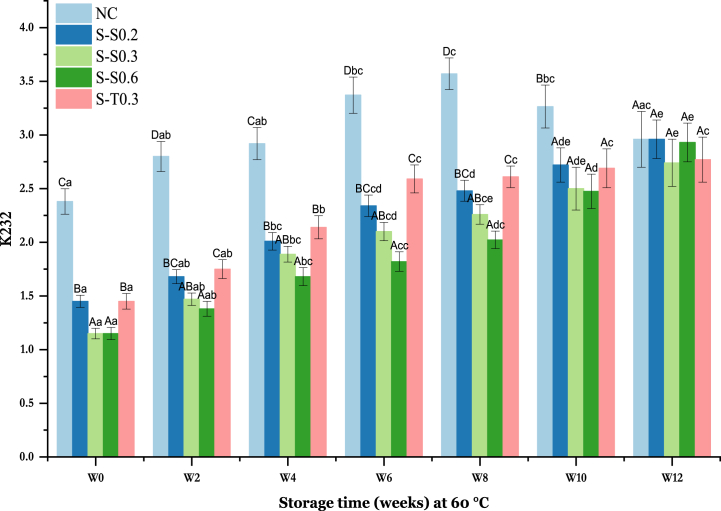
Changes in K232 value of soybean oil (SO) fortified with saffron stigma (SS) during 12 weeks of storage at 60 °C **NC**: Negative control, **S–S0.2**: SO + 0.2% SS, **S–S0.3**: SO + 0.3% SS, **S–S0.6**: SO + 0.6% SS, **S-T0.3**: SO + 0.3% tocobiol. Different letters represent significant differences (p < 0.05) between the oils. The uppercase letters correspond to different treatments for each storage period, while the lowercase letters correspond to the storage periods (W0 to W12) for each treatment.

During the oxidation process, the primary hydroxides (conjugated dienes, CDs) are also revealed by K232 [60,61]. These CDs provide relatively stable chemical markers of polyunsaturated fatty acid oxidation [62]. It is therefore a good variable for the evaluation of oil oxidation [63]. As for PV, the results in Fig. 3 also show that K232 increased according to storage time for all oil samples.

The highest values of K232 were distinctly observed in the negative control (NC) until week 10. The substantial increase in K232 in NC samples indicates a significant formation of primary hydroperoxides during storage. Thus, under the influence of high temperatures, double bonds undergo isomerization, and conjugated systems are formed, leading to the generation of dienes [61]. The K232 of soybean oil containing 0.2, 0.3, and 0.6% SS ranged from 1.45 ± 0.15 to 2.96 ± 0.18, from 1.15 ± 0.12 to 2.74 ± 0.22, and from 1.15 ± 0.08 to 2.93 ± 0.18, respectively. The NC experienced an increase in K232 from 2.38 ± 0.22 to 3.57 ± 0.18 by the 8th week, then, there was a subsequent decrease to 2.96 ± 0.26 by the end of the storage period. The K232 of S-T0.3 (0.3% tocobiol) increased from 1.45 ± 0.14 to 2.77 ± 0.21. This variation shows that enrichment of SO with SS serves as a better antioxidant than tocobiol against oxidative rancidity of refined SO. The obtained results are in agreement with the study conducted by Salaj et al. [64], who examined the oxidative stability of sunflower and olive oils enriched with an organic extract from *Satureja kitaibelii*. They reported that the conjugated dienes of the oil enriched with the extract were lower than those of the control as well as that the extract acts against lipid oxidation. Furthermore, Yang et al. [65], used rosemary extracts to protect refined SO from oxidation compared to synthetic antioxidants (TBHQ, carnosic acid, and rosmarinic acid). They observed that the absorbance of the oils with antioxidants was significantly different from the control and that rosemary extract had the best inhibitory effect on the accumulation of conjugated dienes during frying.

### 3.3 Secondary oxidation

#### 3.3.1 Changes in p-AV

**Fig. 4:**
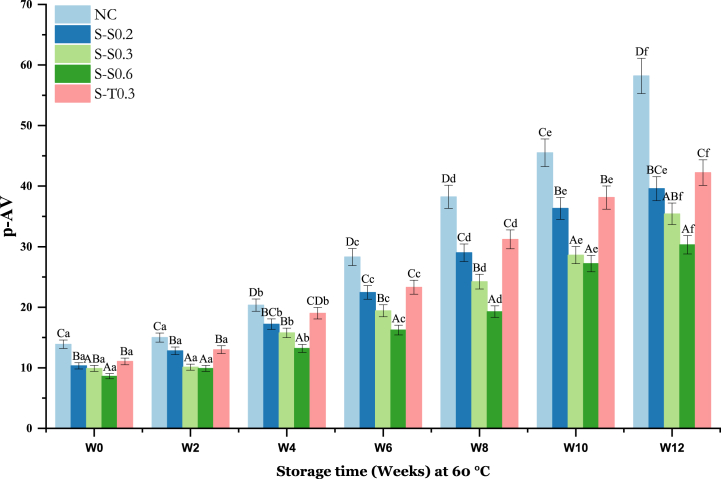
Changes in p-AV of soybean oil (SO) fortified with saffron stigma (SS) during 12 weeks of storage at 60 °C. **NC**: Negative control, **S–S0.2**: SO + 0.2% SS, **S–S0.3**: SO + 0.3% SS, **S–S0.6**: SO + 0.6% SS, **S-T0.3**: SO + 0.3% tocobiol. Different letters represent significant differences (p < 0.05) between the oils. The uppercase letters correspond to different treatments for each storage period, while the lowercase letters correspond to the storage periods (W0 to W12) for each treatment.

PV alone does not reveal the oxidation state of the oil because it is only an indicator of the presence of primary oxidation products and does not indicate the production of secondary products [59]. For this reason, it is appropriate to resort to p-AV for the evaluation of oxidation development. This index indicates the quantity of aldehydic and ketonic hydro-peroxide forms in the oils [54]. It depends on oil type, with a value below 10 indicating good quality of oil [57]. Fig. 4 shows the effect of SS on p-AV values of fortified SO as compared to tocobiol and the NC.

The negative control exhibited the highest p-AV progression, closely, followed by S-T0.3, with the p-AV ranging from 13.89 ± 0.5 to 58.21 ± 0.4 and 11.06 ± 0.9 to 42.43 ± 2.32, 10.34 respectively. The evolution of p-AV in S–S0.2 and S-T0.3 showed a parallel and almost similar pattern throughout the storage period. By the end of the storage period, oils enriched with 0.6% SS and 0.3% SS showed a notable neutralizing effect on the generation of secondary hydroperoxides compared to the negative control and oil enriched with tocobiol 0.3%, this is evidenced by the lower progression of p-AV (9.89 ± 0.83 to 35.42 ± 1.3, and 8.62 ± 0.85 to 30.32 ± 1.60, respectively). The SS added to the oils apparently has an antioxidant effect which may explain this result. A similar result was reported for nano-encapsulated *Ferula persica* polyphenolic extracts, which decreased p-AV over time in SO compared to pure oil after 24 days of storage at 60 °C [66]. In agreement with a previous study, it is shown that methanolic extracts of *Terminalia nigrovenulosa* leaves are more effective in inhibiting secondary oxidation of SO than BHA [67]. The inhibitory effect of SS on p-AV increases in a dose-dependent manner. A similar finding was also reported for *Moringa oleifera* Lam. Leaves extracts which can significantly inhibit SO oxidation and whose inhibitory effect increases as their concentration increases [68].

#### 3.3.2 Changes in K270

**Fig. 5:**
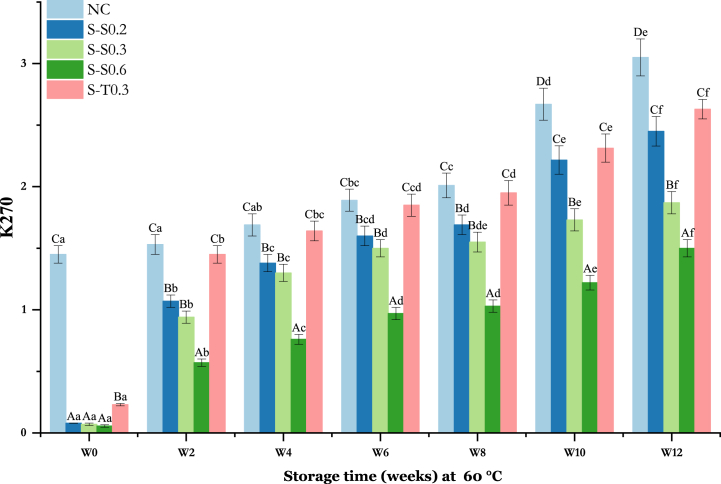
Changes in K270 value of soybean oil (SO) fortified with saffron stigma (SS) during 12 weeks of storage at 60 °C. **NC**: Negative control, **S–S0.2**: SO + 0.2% SS, **S–S0.3**: SO + 0.3% SS, **S–S0.6**: SO + 0.6% SS, **S-T0.3**: SO + 0.3% tocobiol. Different letters represent significant differences (p < 0.05) between the oils. The uppercase letters correspond to different treatments for each storage period, while the lowercase letters correspond to the storage periods (W0 to W12) for each treatment.

K270 measures the presence of conjugated trienes (CTs), aldehydes, ketones and primary oxidation products of linolenic acid [69,70]. An increase in K270 of the oil samples was observed for 12 weeks. However, the unenriched samples had a considerably higher K270 than the enriched samples. According to Fig. 5, it is evident that the initial K270 level was higher for NC (1.45 ± 0.12) (p < 0.05), followed by S-T0.3 (0.23 ± 0.11). Meanwhile, saffron-enriched oils S–S0.2, S–S0.3, and S–S0.6 had roughly equal initial K270 values, with values of 0.078 ± 0.09, 0.073 ± 0.06 and 0.058 ± 0.06, respectively.

At time zero, the initial findings revealed significantly higher value (p < 0.05) for the negative control (1.45 ± 0.07) in comparison to the oil enriched with tocobiol (S-T0.3, 0.23 ± 0.03). The latter, in turn, demonstrated a significantly higher value (p < 0.05) than oils enriched with saffron at various concentrations. Following the initial two weeks of storage, the generation of secondary oxidation products increased significantly in oils containing antioxidants, particularly for S-T0.3 (from 0.23 ± 0.11 to 1.45 ± 0.16). Indeed, an analogous increase was observed in the negative control in this period (2 weeks of storage), demonstrating a non-significant rise (p > 0.05) when compared to the oil enriched with tocobiol (S-T0.3). Remarkably, the oil enriched with 0.6% saffron concentration exhibited the lowest evolution among the samples (p < 0.05). The values of conjugated trienes displayed an almost linear increase from 2 to 10 weeks across all samples. By the 10th week, the values reached high levels for the negative control (2.67 ± 0.10), the tocobiol-enriched oil (S-T0.3, 2.67 ± 0.10), and the saffron-enriched oil at a concentration of 0.2% (S–S0.2, 2.21 ± 0.09). This trend continued until week 12, consistently marking the lowest evolution in the oil enriched with 0.6% saffron concentration (S–S0.6). The observed increase in K270 in this study can be assigned to the conversion of primary oxidation products into secondary products. These results are also confirmed by those obtained for the p-AV. Abd-El-hady et al. [71], studied the oxidative stability of sunflower and SO, to which phenolic extracts of red onion scale (ROS) and potato peel (PP) and BHT were added at 65 °C. They reported that the CTs of the oils added with natural extracts (ROS and PP) at 900 ppm were lower than the control and that these extracts act against lipid oxidation. It is thus found that the inhibition efficiency of CTs for treated sunflower oil corresponds to the order ROS900 > PP900 > ROS600 > PP600 > BHT200 > ROS300 > PP300 and that of treated soybean corresponds to ROS900 > PP900 > ROS600 > PP600 > BHT200 > ROS300 > PP300.

### 3.4 Changes in TOTOX

TOTOX represents an index to evaluate the overall oxidation level of the oil [68]. It reflects the primary and secondary stages of oxidation, thus allowing a better evaluation of the progressive oxidative deterioration of oils [42,72].

**Fig. 6:**
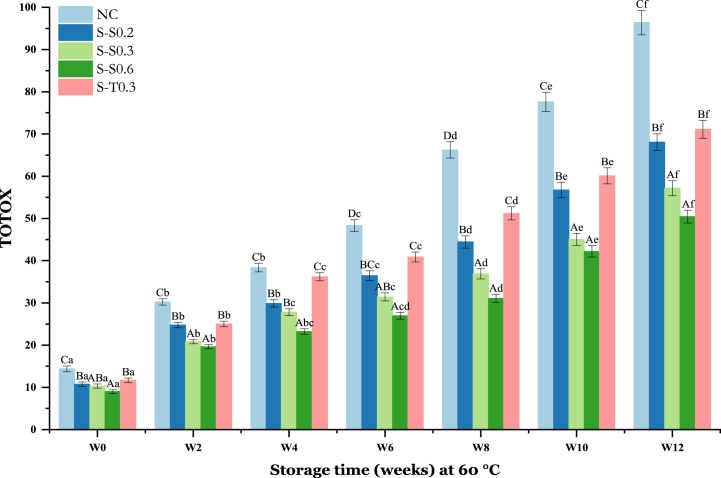
Changes in TOTOX value of soybean oil (SO) fortified with saffron stigma (SS) during 12 weeks of storage at 60 °C. **NC**: negative control, **S–S0.2**: SO + 0.2% SS, **S–S0.3**: SO + 0.3% SS, **S–S0.6**: SO + 0.6% SS, **S-T0.3**: SO + 0.3% tocobiol. Different letters represent significant differences (p < 0.05) between the oils. The uppercase letters correspond to different treatments for each storage period, while the lowercase letters correspond to the storage periods (W0 to W12) for each treatment.

The TOTOX value followed a similar trend as PV and p-AV values, exhibiting a sharp increase in the first week followed by a linear rise with extended storage time. Nevertheless, oils lacking antioxidants (NC) demonstrated the highest TOTOX values in comparison to the fortified oil samples over the storage duration (Fig. 6). These values increase from 14.39 ± 2.79 at the initial time to 30.23 ± 2.98 in the second week, and subsequently tripled to 96.39 ± 3.50 by the week 12. This indicates greater chemical deterioration of these NC oils due to exposure to heating at 60 °C. TOTOX of SO samples containing 0.2, 0.3, and 0.6% SS increased from 10.74 ± 1.42 to 68.68 ± 3.33, 10.31 ± 1.33 to 57.18 ± 2.33, 9 ± 1.77 to 50.42 ± 0.9 at the end of the storage period, respectively. The samples containing tocobiol (S-T0.3) closely resembled to soybean oil containing 0.2% SS (S–S0.2) at the initial time (first week). However, by the end of the 12-week period, the values of samples containing tocobiol (S-T0.3) increased significantly (p < 0.05) to 71.11, exceeding the values of SS-enriched oils, suggesting a greater stability of the latter than tocobiol. The stability order was S–S0.6 > S–S0.3 > S–S0.2 > S-T0.3 > NC. TOTOX was effectively reduced in oil samples amended with SS due to the antioxidant compounds they contain such as crocin, crocetin, safranal, etc [73]. Therefore, SS can be a potential source of bioactive extracts for the development of bioactive ingredients and to produce functional foods [73]. Hassan et al. [55], also confirmed the better antioxidant efficacy of thyme extracts (presented lower TOTOX value) in preventing SO oxidation compared to the synthetic antioxidant TBHQ. This effectiveness is also observed for the incorporation of the essential oil of *Punica granatum cv. Heyinshiliu* at 800 ppm which is able to inhibit the increase of TOTOX in sunflower oil heated at 65 °C for 30 days [72].

### 3.5 Changes in FA

Fatty acid composition is an important indicator of the stability and nutritional value of foods [[74], [75], [76]]. The FA can make oils sensitive to oxidation, the existence of two double bonds in the structure of fatty acids making oxidation 10 to 40 times faster than with a single double bond [8]. It is an estimate of oil lipolysis [63]. The FA of SO was determined at time zero for the control and the fortified oils as well as at the final time after storage at 60 °C for 12 weeks and the results are presented in Table 1.

**Table 1:** Changes in fatty acid composition (g/100 g) and iodine value (g I_2_/100 g) of soybean oil (SO) during 12 weeks of storage at 60 °C.

	NC		S–S0.2		S–S0.3		S–S0.6		S-T0.3	
Initial (W0)	Final (W12)	Initial (W0)	Final (W12)	Initial (W0)	Final (W12)	Initial (W0)	Final (W12)	Initial (W0)	Final (W12)	
Palmitic acid C16:0	11.32^Aa^±0.05	12.38^Ab^±0.10	11.14^Aa^±0.11	11.22^Aa^±0,21	11.06^Aa^±0.05	11.22^Aa^±0.50	11.08^Aa^±0.10	11.12^Aa^±0.17	11.19^Aa^±0.10	11.22^Aa^±0.30
stearic acid C18:0	4.82^Aa^±0.22	5.44^Ab^±0.02	5.21^Aab^±0.10	5.26^Ab^±0.07	5.11^**A**ab^±0.10	5.21^Ab^±0.09	5.05^Aab^±0.20	5.26^Ab^±0.10	5.09^Aab^±0.20	5.37^Ab^±0.10
Oleic acid C18:1	22.21^Aab^±0.20	21.65^Aa^±0.05	22.37^Ab^±0.05	22.54^Bb^±0.05	22.48^Ab^±0.10	22.39^ABb^±0.19	22.25^Ab^±0.03	22.12^ABab^±0.07	22.26^Ab^±0.52	22.39^ABb^±0.21
Linoleic acid C18:2	54.17^Ab^±0.07	53.41^Aa^±0.20	54.21^Ab^±0.21	54.18^Ab^±0.05	54.10^Ab^±0.10	54.04^Aab^±0.19	54.14^Ab^±0.50	54.08^Ab^±0.10	54.23^Ab^±0.13	54.09^Ab^±0.31
Linolenic acid C18:3	7.13^Aa^±0.07	6.73^Aa^±0.10	6.74^Aa^±0.90	6.48^Aa^±0.50	6.91^Aa^±0.05	6.82^Aa^±0.10	7.14^Aa^±0.10	7.11^Aa^±0.17	6.87^Aa^±0.50	6.61^Aa^± 0.21
Arachidic acid C20:0	0.35^Aa^±0.03	0.39^Aa^±0.02	0.33^Aa^±0.05	0.32^Aa^±0.04	0.34^Aa^±0.05	0.32^Aa^±0.05	0.34^Aa^±0.07	0.31^Aa^±0.02	0.36^Aa^±0.07	0.32^Aa^±0.03
SFA	16.49^Aa^±0.1	18.21^Bc^±0.05	16.68^Aab^±0.09	16.80^Aab^±0.11	16.51^Aa^±0.07	16.75^Aab^±0.21	16.47^Aa^±0.12	16.69^Aab^±0.10	16.64^Aab^±0.12	16.91^Ab^±0.14
MUFA	22.21^Aab^±0.20	21.65^Aa^±0.05	22.37^Ab^±0.05	22.54^Bb^±0.05	22.48^Ab^±0.10	22.39^ABb^±0.19	22.25^Ab^±0.03	22.12^ABab^±0.07	22.26^Ab^±0.52	22.39^ABb^±0.21
PUFA	61.30^Ab^±0.07	60.14^Aa^±0.15	60.95^Ab^±0.56	60.66^Aab^±0.22	61.01^Ab^±0.05	60.86^Ab^±0.15	61.28^Ab^±0.30	61.19^Ab^±0.14	61.10^Ab^±0.32	60.70^Aab^±0.26
Iodine Value (g (I_2_)/100 g)	137.75^Ab^±0.29	134.77^Aa^±0.65	136.89^Aa^±0.39	136.28^Aa^±0.23	137.26^Aa^±0.49	136.82^Aa^±1.3	137.75^Aa^±0.16	137.45^Aa^±1.5	137.19^Aa^±0.29	136.34^Aa^±0.67

SFA: Saturated Fatty Acids MUFA: Monounsaturated Fatty Acids PUFA: Polyunsaturated Fatty Acids.

**NC**: negative control, **S–S0.2**: SO + 0.2% SS, **S–S0.3**: SO + 0.3% SS, **S–S0.6**: SO + 0.6% SS, **S-T0.3**: SO + 0.3% tocobiol. The data are reported as the average of triplicates (n = 3). The uppercase letters correspond to different treatments for each storage period, while the lowercase letters correspond to the storage periods (W0 to W12) for the same treatment.

Polyunsaturated fatty acids (PUFA) occupy a predominant place in SO fatty acid, with an average value of 61.30 g/100 g, followed by monounsaturated fatty acids (MUFA) with a content of 22.21 g/100 g, and a level of 16.49 g/100 g of saturated fatty acids (SFA). Linoleic acid (C18:2) was the predominant PUFA and oleic acid (C18:1) was the most abundant MUFA at 54.17 and 22.21 g/100 g, respectively. The SFA were dominated by palmitic (C16:0) and stearic acid (C18:0) with levels of 11.32 and 4.82 g/100 g, respectively. This finding is in a harmony with that obtained by Franco et al. [63].

With respect to the negative control, our results show that MUFA and PUFA decreased markedly from 22.21 to 21.65 g/100 g and from 61.30 to 60.14 g/100 g, respectively. While SFA increased from 16.49 to 18.21 g/100 g. In the SS-fortified oils, a marginal change was obtained regarding the MUFA level, from 22.37 to 22.54 for S–S0.2, from 22.48 to 22.39 for S–S0.3, and from 22.25 to 22.12 g/100 g for S–S0.6. The changes in the PUFA were from 60.95 to 60.66 for S–S0.2, from 61.01 to 60.86 for S–S0.3 and from 61.28 to 61.19 g/100 g for S–S0.6. Fortifying the oil with the tocobiol, increased the MUFA by 0.58% and decreased the PUFAs by 0.65%.

After 12 weeks' storage at 60 °C, the fatty acid composition of the negative control (NC) experienced notable changes, with reductions of 2.52, 1.40, and 5.94% observed for oleic, linoleic, and linolenic acids, respectively. A reduction of linoleic (− 0.26%) and linolenic acids (− 3.78%) was also observed in tocobiol-enriched oils, while an increase of oleic acid (+0.58%) was registered. These PUFA were substantially reduced by oxidation reactions during the thermal processes. PUFA are notoriously sensitive to oxidation [77]. The incorporation of SS into SO samples delayed the degradation of unsaturated fatty acids (linoleic and linolenic acid) compared to control and tocobiol samples. Furthermore, a relatively low degradation rate of these unsaturated acids was noticed in S–S0.6, which reduced oleic acid by (− 0.58%), linolenic acid by (− 0.42% and linoleic acid by (− 0.11%). A similar result was found by Yang et al. [78], at 62 °C for 24 days, wherein the incorporation of rosemary extract into the oils preserved the integrity of unsaturated fatty acids (C18:1, C18:2 and C18:3) and a similar trend was observed for synthetic antioxidants (BHQ and BHT) but with a lower efficiency. Huang et al. [79], carried out a fatty acids profiling of soybean oil fortified with 200 ppm of quercetagetin QG (a bioactive compound present in the forage plant), propyl gallate (propyl 3,4,5-trihydroxybenzoate, PG), BHT, and tea polyphenols. They revealed that QG significantly inhibited the oxidation of the unsaturated fatty acids linoleic acid (C18:2) and oleic acid (C18:1).

### 3.6 Changes in IV

IV is a measure of the degree of unsaturation of oils and fats [[80], [81], [82]]. It determines oil stability to oxidation [83]. The initial IV of all oils were almost identical (137 g I_2_/100 g oil) with the exception of S–S0.2 which had an IV of 136.89 g I_2_/100 g oil (Table 1). This high value of IV is due to high content of unsaturated fatty acids essentially due to the abundance of oleic and linoleic acids. IV tended to decrease throughout the storage period. Notably, a noticeable reduction was observed in the soybean oil in the case of the negative control (NC), decreasing from 137.75 ± 0.29 to 134.77 ± 0.65 g I_2_/100 g of. This trend aligns with findings presented by Abd-Allah et al. [51], where control oils displayed a higher rate of IV reduction compared to oils containing synthetic (BHT) and natural (pomegranate and orange peel extract) antioxidants after 24 days of storage at 65 °C. The oil samples treated with the synthetic antioxidant tocobiol (S-T0.3) exhibited the second most substantial reduction in IV by 0.85 g I_2_/100 g (from 137.19 ± 0.29 to 136.34 ± 0.67 g I_2_/100 g), then those treated with 0.2% SS (S–S0.2) experienced a reduction of 0.61 g I_2_/100 g (from 136.89 ± 0.39 to 136.82 ± 1.3 g (I_2_)/100 g). Both S–S0.3 and S–S0.6 had the lowest rate of decrease in IV after the storage period, 0.44 and 0.31 g I_2_/100 g, respectively, suggesting that unsaturated fatty acids were less oxidized in the oil samples mixed with SS. These results are confirmed by the treatment of SO with *Moringa oleifera* seed oil [84], banana, mango and orange peels extracts [85]. Likewise, Olagunju et al. [83], showed that the incorporation of rice bran extracts at 500 and 1000 ppm results in a 4.5% decrease in the IV of the crude SO. More recently, Oubannin et al. [86], observed a similar trend of IV variation for argan [*Argania spinosa* L. (Skeels)] oil extracted mechanically with thyme (*Thymus vulgaris* L.) leaves. The performance of SS to inhibit oil oxidation was highly dose-dependent. However, all SS doses used (0.2, 0.3, and 0.6%) were effective as compared to the positive (tocobiol at 0.3%) and the negative control (no enrichment). SS with their enormous health-promoting properties [87], let encourage their consideration by vegetable oil industry as a potential natural antioxidant stabilizer as suggested by Gharby et al. [88].

### 3.7 Multivariate analysis

#### 3.7.1 Correlation and simple regression study

**Fig. 7:**
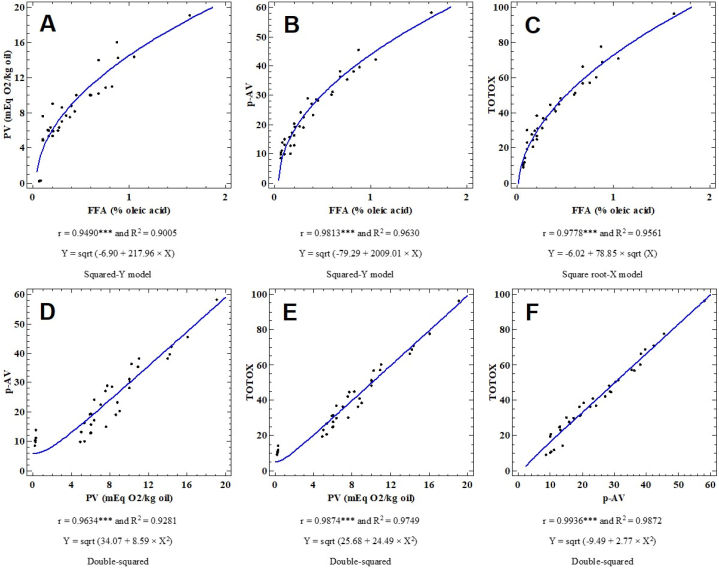
Simple regression models between some basic quality indices measured in soybean oil enriched with various doses of saffron stigma. Such indices are free fatty acids (FFA), anisidine value (p-AV), peroxide value (PV), UV extinction coefficients K232 and K270, as well as total oxidation value (TOTOX). R^2^: determination coefficient, r: correlation coefficient, and sqrt: square root. *** indicate significance at the 0.001 probability level. The points plotted are mean values averaged over storage times and enricher doses.

Table 2 depicts the correlation matrix among routinely measured quality indices determined each two weeks throughout the storage period. As can be seen in these results, very highly significant positive associations were revealed among the studied quality indices. Very highly significant correlations with a determination coefficient (R^2^) exceeding 0.90 were modeled through simple regressions, which were performed on mean values over storage time and enricher dose (Fig. 7). Peroxide value was regressed on free fatty acids (Fig. 7A), the best fitted model was of square root type (Eq. (4)).

(4) $$Y=\sqrt{a\times X+b}/\;\left(a,b\right)\;\epsilon \;R²\;\text{with}\;R2=0.9005$$

**Table 2:** Correlation (Pearson) coefficients among routinely measured quality indices namely free fatty acids (FFA), anisidine value (p-AV), peroxide value (PV), UV extinction coefficients K232 and K270, as well as total oxidation value (TOTOX). *** indicates significance level at p < 0.001.

	FFA	p-AV	PV	K232	K270	TOTOX
**FFA**		0.9631***	0.8977***	0.685***	0.8427***	0.9545***
**p-AV**			0.9181***	0.8073***	0.8885***	0.9849***
**PV**				0.8213***	0.9170***	0.9728***
**K232**					0.8532***	0.8300***
**K270**						0.9193***
**TOTOX**

Similarly, *p*-anisidine value was regressed separately on free fatty acids (Fig. 7B, R^2^ = 0.9630) and peroxide value (Fig. 7D, R^2^ = 0.9281). The corresponding models were square root and double squared (Eq. (5)) as the best fit models, respectively.

(5) $$Y=\sqrt{a\times X^{2}+b}/\;\left(a,b\right)\;\epsilon \;R²$$

TOTOX as the total oxidation value was regressed on free fatty acids (Fig. 7C, R^2^ = 0.9561), peroxide value (Fig. 7E, R^2^ = 0.9749), and *p*-anisidine value (Fig. 7F, R^2^ = 0.9872). The corresponding best fit models were square-root X model (Eq. (6)) for TOTOX versus FFA and double-squared for both TOTOX versus PV and TOTOX versus p-AV.

(6) $$Y=a\sqrt{X}+b/\;\left(a,b\right)\;\epsilon \;R²$$

In literature, several studies performed on vegetable edible oils have highlighted similar trends of correlations among basic quality indices. Following these authors, FFA have a prooxidant effect on vegetable oils. Besides, the formation of FFA is related to hydroperoxides content and oxidation of aldehydes, which explain the strong positive association between FFA and PV. Indeed, oil oxidation gives rise to products like hydroperoxides and their corresponding derivatives (measured as FFA and PV) are conjugated dienes and trienes. These compounds absorb at λ = 232 and λ = 270 nm, respectively. Hydroperoxides (primary oxidation products) absorb light at 232 nm (K232) and they are unstable and tend to be quickly converted into secondary oxidation products (mostly diketones as well as unsaturated ketones) evaluated through as p-AV. Such secondary oxidation compounds absorb light at 270 nm (K270). This could the reason behind the positive associations among K232, K270, p-AV, and FFA.

#### 3.7.2 Principal component analysis

**Fig. 8:**
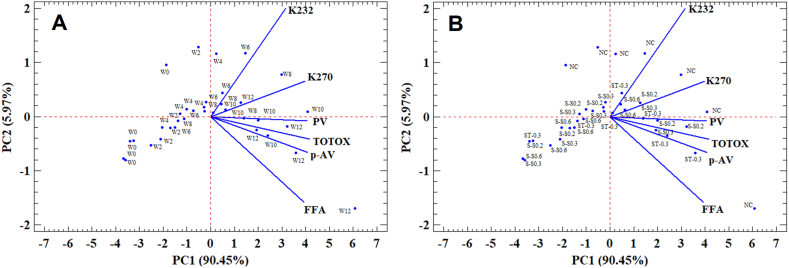
Principal component analysis (PCA) projections on the first (PC1) and the second component (PC2). The eigenvalues are presented as blue segments showing variables that most affect each principal component. The points plotted are storage time mean values (A) and enricher dose mean values (B) of soybean oil quality indices namely fatty acids (FFA), anisidine value (p-AV), peroxide value (PV), UV extinction coefficients K232 and K270, as well as total oxidation value (TOTOX). S–S0.2, S–S0.3, and S–S0.6 are soybean oil fortified with saffron stigma at 0.2, 0.3, and 0.6%, respectively. S-T0.3: Soybean oil +0.3% tocobiol (positive control) and NC: Negative control (soybean oil without any additives). W0 to W12: Represent storage time in weeks (W). (For interpretation of the references to color in this figure legend, the reader is referred to the Web version of this article.)

As shown in Table 3, 5 principal components (PC) were extracted accounting for 100% as the total variance. Furthermore, the PCA outcomes show that the first two PCs explained over 96% of the total observed variance. PC1 and PC2 accounted for about 90.45%, and 5.97% from the total variability, respectively. The points plotted on the surface delimited by PC1 and PC2 (Fig. 8A) are related to storage times.

**Table 3:** The extracted principal components, their eigenvalue, percent of variance, and cumulative percentage.

Component number	Eigenvalue	Percent of variance	Cumulative percentage (%)
1	05.43	90.454	90.454
2	35.84 × 10^−2^	5.973	96.427
3	11.76 × 10^−2^	1.959	98.386
4	76.78 × 10^−3^	1.280	99.666
5	20.04 × 10^−3^	0.334	100.000

As depicted in Fig. 8A, PC1, with its associated variance exceeding 90%, seems to separate storage times. Toward its negative part, were distributed the points associated to the first storage weeks mainly W0, W2, and W4 with relatively low scores of FFA, PV, p-AV, K232, K270, and TOTOX. In contrast, on the PC1 positive side, were distributed the points linked to W6, W8, W10, and W12 marked by higher values of the quality indices. Similarly, points associated to enricher dose together with the negative (NC) and the positive control (S-T0.3: soybean oil + 0.3% tocobiol) were distributed along the PC2 (Fig. 8B). The lowest dose of enricher as well as NC were associated to high levels of FFA, PV, p-AV, K232, K270, and TOTOX. However, higher enricher doses (S–S0.3 and S–S0.6) along with the positive control S-T0.3 were marked by relatively low values of the plotted quality indices. Similar evidences were found in literature on the use of PCA as a multivariate approach to reduce data dimensionality and investigate relationship among different dependent variables and factors in edible vegetable oils and their enrichment using natural antioxidants recovered from plant matrices [[89], [90], [91], [92]].

## 4 Conclusions

Saffron stigmas were highly effective in stabilizing soybean oil across all concentrations, particularly at 0.6%, showcasing a robust antioxidant effect during both the initial and final stages of oxidation throughout various storage periods. Saffron stigmas at concentrations of 0.3% and 0.2% consistently demonstrate more or less significant antioxidant effects compared with the synthetic antioxidant used (tocobiol). Fatty acid composition indicates that the incorporation of these antioxidants does not alter the distribution of fatty acids in soybean oils. Therefore, we can see that the antioxidant activity of saffron stigmas was generally superior to that of the synthetic antioxidant (tocobiol) given their long-term effectiveness and consistent protection. Furthermore, saffron stigmas have good potential to be studied as a source of natural antioxidants and may be preferred over synthetic antioxidants such as tocobiol as a stabilizer for table oils. In short, the results of the present study allow to motivate a greater attention to natural substances in the field of food additives, as one of the most effective solutions for the stability of vegetable oils.

## Data availability statement

The data that support the findings of this study are available from the corresponding authors, E.H. Sakar and S. Gharby, upon a reasonable request.

## Additional information

No additional information is available for this paper.

## CRediT authorship contribution statement

**Moussa Nid Ahmed:** Writing – original draft, Software, Methodology, Conceptualization. **Karima Abourat:** Formal analysis, Data curation. **Jamila Gagour:** Writing – original draft, Resources, Investigation, Formal analysis. **El Hassan Sakar:** Writing – review & editing, Validation, Software, Resources, Conceptualization. **Khalid Majourhat:** Writing – original draft, Resources, Data curation. **Jamal Koubachi:** Resources, Formal analysis. **Said Gharby:** Writing – review & editing, Validation, Supervision, Project administration, Conceptualization.

## Declaration of competing interest

The authors declare that they have no known competing financial interests or personal relationships that could have appeared to influence the work reported in this paper.
